# Trend changes in age-related body mass index gain after coronavirus disease 2019 pandemic in Japan: a multicenter retrospective cohort study

**DOI:** 10.1186/s12958-023-01061-1

**Published:** 2023-01-19

**Authors:** Sho Tano, Tomomi Kotani, Takafumi Ushida, Yukako Iitani, Kenji Imai, Fumie Kinoshita, Hiroaki Kajiyama

**Affiliations:** 1 Department of Obstetrics and Gynecology, Nagoya University Graduate School of Medicine, Nagoya, Aichi Japan; 2 Division of Perinatology, Center for Maternal-Neonatal Care, Nagoya University Hospital, 65 Tsurumai-Cho, Showa-Ku, Nagoya, Aichi 466-8560 Japan; 3 Data Science Division, Data Coordinating Center, Department of Advanced Medicine, Nagoya University Hospital, Nagoya, Aichi Japan

**Keywords:** Age-related weight gain, Worker, COVID-19, Obesity, Lockdown, Diet

## Abstract

**Background:**

Accumulating studies suggest that strict lockdown with enforcement including segregation to control the coronavirus disease 2019 (COVID-19) pandemic is associated with excess weight gain, but the such lockdown was not practiced in Japan. We aimed to compare the age-related weight gain before and after the COVID-19 pandemic in Japan where achieved epidemic control based on individual voluntary action.

**Methods:**

This multicenter retrospective cohort study used electronic data from annual health checkups for workers from January 2015 to December 2021 at four facilities belonging to the Central Clinic Group, Aichi, Japan. We defined pre-pandemic and post-pandemic periods as January 2015–December 2019 and January 2020–December 2021, respectively. Participants were grouped by sex, age, and body mass index (BMI) stratus as of 2015, and the pre-pandemic and post-pandemic age-related BMI changes in overall individuals and each specific group were compared using a paired *t*-test.

**Results:**

The total number of eligible participants was 19,290. During the pre-pandemic period, the mean BMI increased linearly in every group. The mean age-related BMI changes in females’ pre-pandemic and post-pandemic periods were + 0.11 and + 0.02 kg/m^2^/year, respectively. This significant decrease was also shown in males, + 0.11 in the pre-pandemic and − 0.02 kg/m^2^/year in the post-pandemic periods. The reduction was consistently observed in all age strata. Furthermore, a significant reduction was also observed in the normal-weight females of reproductive ages aged 15–44 years.

**Conclusions:**

This is the first report showing that age-related weight gain was reduced after the COVID-19 pandemic in Japan, which could affect the reproductive age of females.

**Supplementary Information:**

The online version contains supplementary material available at 10.1186/s12958-023-01061-1.

## Background

Being overweight, particularly weight gain in early adulthood, is a common risk factor for major health outcomes [1. ]. The body mass index (BMI) increases with age [2. ], and this age-related BMI gain is associated with several diseases [3. –6. ]. Many guidelines discussing weight management seem to assume that weight can be maintained. However, we have previously discussed the importance of setting goals considering that body weight increases with age and stated that it is important to determine how much weight gain is acceptable [4. –6. ]. There have been increasing concerns regarding the effect of the coronavirus disease 2019 (COVID-19)-related lockdowns on body weight or BMI worldwide. A recent meta-analysis sounded the alarm that the lockdowns to control the COVID-19 pandemic would result in a higher incidence of overweight or obesity, leading to related health problems [7. ]. Most studies were conducted in regions with strict lockdown enforcement, including segregation [8. ]. However, the epidemic control measures implemented in Japan were quite different from such strict lockdown enforcement. Japan had promoted warning against the ‘3Cs’: closed environments, crowded conditions, and close-contact settings since January 15, 2020, when the first case was reported [9. ] and achieved epidemic control based on individual voluntary action not to eat or drink out [10. –12. ]. A recent study proposed that public health policies modify the effect on physical activity or dietary habits [13. ], which are ultimately associated with age-related BMI gain. However, to our knowledge, these analyses have not been reported in the Japanese population. This study aimed to compare the situation before and after the COVID-19 pandemic in Japan and discussed the differences.

## Material and methods

**Fig. 1:**
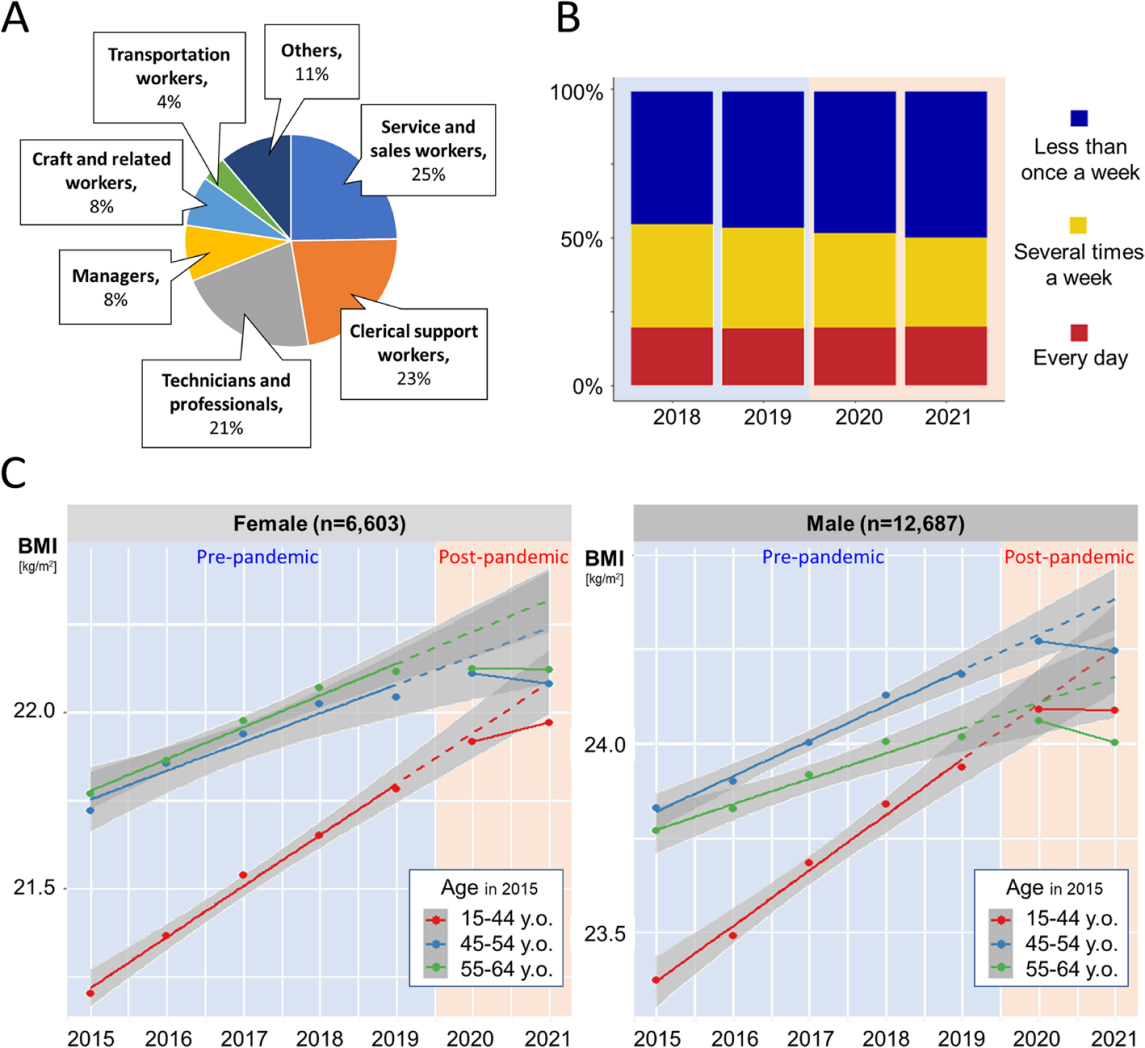
**A** Participants’ job categories. **B** Frequency of drinking between 2018 and 2021. The horizontal and vertical axes of the stacked bar graph represent years and the frequency, respectively. Blue, yellow, and red parts represent less than once a week, several times a week, and every day groups, respectively. **C** BMI change between 2015 and 2021. Scatter plots of mean BMI with regression lines for each age group. The regression lines (solid lines) are based on the measured BMI in the pre-pandemic period (2015–2019) and post-pandemic period (2020–2021); dashed lines are extensions of the former. Shaded areas denote 95% confidence intervals

This multicenter retrospective cohort study was conducted using electronic data from annual health checkups for workers, based on Japanese Law, Industrial Safety and Health Act, from January 2015 to December 2021 at four facilities belonging to the Central Clinic Group, Aichi, Japan. Eligible participants were workers aged 15–64 years in 2015 who had undergone annual health checkups from 4518 companies: their occupational categories are shown in Fig. 1A. The pre-pandemic and post-pandemic periods were defined as January 2015–December 2019 and January 2020–December 2021, respectively.

The alcohol consumption frequency was calculated based on the questionnaire with three choices: every day, several times a week, and less than once a week (Fig. 1B). Female participants were divided according to their life-stage strata (reproductive age, 15–44 years; peri-menopausal age, 45–54 years; and post-menopausal age, 55–64 years) or BMI strata (overweight/obesity, ≥25.0; normal weight, 18.5–25.0; and underweight, < 18.5 kg/m^2^) as of 2015. Male participants also were stratified by the same with age and BMI. The mean measured BMI for each year was calculated for each stratum. The mean age-related BMI change in the pre-pandemic and post-pandemic periods was calculated as the mean annual BMI change within each period and was compared using a paired *t*-test. The data used in the pre-pandemic and post-pandemic periods were from the ‘same’ individuals. A linear regression analysis was performed on the decrease in the mean age-related BMI gain from the pre-pandemic period to the post-pandemic period, with age, sex, and BMI as of 2015 as covariates. Statistical significance was set at two-tailed *p* < 0.05. In cases of multiple comparisons, a Bonferroni correction was applied. Statistical analyses were performed using SPSS version 28.0 for Windows (IBM Corp. Armonk, NY, USA) or R ver.4.1.3 (https://cran.r-project.org/).

## Results

The total number of eligible participants was 19,290: females, *n* = 6603, and males, *n* = 12,687 (Table 1). Service and sales workers, clerical support workers, and technicians and professionals accounted for 25%, 23%, and 21% of the participants, respectively (Fig. 1A). As for the frequency of alcohol consumption, “several times a week” decreased from 34.2% in 2019 to 30.4% in 2021, and “less than once a week” increased from 46.4% in 2019 to 49.6% in 2021 (Fig. 1B). The first state of emergency in Aichi Prefecture was declared on April 10, 2020, and since then, restaurants and bars were requested to close for a total of 28 days in 2020 and 160 days in 2021. There were few enforceable measures, but most restaurants cooperated with the requests.

**Table 1:** Age-related BMI gain in the pre-pandemic and post-pandemic period

Characteristics	n (%)	Age-related BMI gain, kg/m^2^/year		*p*-value
		Pre-pandemic (2015–2019)	Post-pandemic (2020–2021)	
**Female**				
**Overall**	**6603 (100)**	**0.11 ± 0.00**	**0.02 ± 0.01**	**< 0.001**
**15–44 years old**	**3349 (50.7)**	**0.15 ± 0.01**	**0.06 ± 0.01**	< 0.001*
Overweight or obesity	410 (6.2)	0.18 ± 0.03	0.06 ± 0.06	0.100
Normalweight	2269 (34.4)	0.14 ± 0.01	0.05 ± 0.02	< 0.001^§^
Underweight	670 (10.1)	0.14 ± 0.01	0.08 ± 0.02	0.039
**45–54 years old**	**2419 (36.6)**	**0.08 ± 0.01**	**− 0.03 ± 0.02**	< 0.001*
Overweight or obesity	378 (5.7)	0.11 ± 0.02	− 0.01 ± 0.06	0.071
Normalweight	1681 (25.5)	0.08 ± 0.01	− 0.03 ± 0.02	< 0.001^§^
Underweight	360 (5.5)	0.08 ± 0.01	− 0.05 ± 0.03	< 0.001^§^
**55–64 years old**	**835 (12.6)**	**0.09 ± 0.01**	**− 0.02 ± 0.03**	0.002*
Overweight or obesity	120 (1.8)	0.07 ± 0.04	0.03 ± 0.09	0.732
Normalweight	615 (9.3)	0.09 ± 0.01	−0.01 ± 0.03	0.002^§^
Underweight	100 (1.5)	0.10 ± 0.02	−0.01 ± 0.05	0.065
**Male**				
**Overall**	**12,687 (100)**	**0.11 ± 0.00**	**−0.02 ± 0.01**	**< 0.001**
**15–44 years old**	**6573 (51.8)**	**0.14 ± 0.01**	**0.00 ± 0.01**	< 0.001*
Overweight or obesity	1746 (13.8)	0.09 ± 0.01	− 0.04 ± 0.03	< 0.001^§^
Normalweight	4442 (35.0)	0.16 ± 0.00	0.01 ± 0.01	< 0.001^§^
Underweight	385 (3.0)	0.18 ± 0.01	0.04 ± 0.03	< 0.001^§^
**45–54 years old**	**4180 (32.9)**	**0.09 ± 0.00**	**− 0.03 ± 0.01**	< 0.001*
Overweight or obesity	1300 (10.2)	0.05 ± 0.01	− 0.03 ± 0.03	0.006
Normalweight	2754 (21.7)	0.10 ± 0.01	− 0.02 ± 0.01	< 0.001^§^
Underweight	126 (1.0)	0.16 ± 0.02	− 0.03 ± 0.06	0.003^§^
**55–64 years old**	**1934 (15.2)**	**0.06 ± 0.01**	**−0.06 ± 0.02**	< 0.001*
Overweight or obesity	610 (4.8)	0.01 ± 0.01	− 0.11 ± 0.04	< 0.001^§^
Normalweight	1270 (10.0)	0.08 ± 0.01	− 0.03 ± 0.02	< 0.001^§^
Underweight	54 (0.4)	0.16 ± 0.04	−0.07 ± 0.08	0.008

Age-related BMI gain was expressed as mean ± standard error (SE) and *p*-value for the paired t-test

*BMI* Body mass index, *n* Numbers in each group

*p-values were considered significant at < 0.0167 (Bonferroni correction: 0.05/3 = 0.0167)

^§^ *p*-values were considered significant at < 0.0056 (Bonferroni correction: 0.05/9 = 0.0056)

In every sex and age group, the BMI increased linearly in the pre-pandemic period, but the inclination of the graph, corresponding to age-related BMI gain, abruptly decreased after the pandemic, except for the 15–44 years of the female group (Fig. 1C). The actual BMI increment was smaller than expected, even in the 15–44 years female group. As for weight loss in the 15–44 years old group (Table S1), the highest frequency of weight loss was observed in 2021 for both sexes (43.6% for females and 47.4% for males).

As shown in Table 1, the overall mean age-related BMI gain in both sexes in the pre-pandemic period was + 0.11 kg/m^2^/year. Female participants of reproductive age (15–44 years of age) showed the highest mean age-related BMI gain: the values were 0.15 kg/m^2^/year, 0.08 kg/m^2^/year, and 0.09 kg/m^2^/year for those aged 15–44, 45–54, and 55–64 years, respectively. A similar tendency was also observed in the respective age groups of male participants.

The overweight or obesity group of females had the highest age-related BMI gain in the reproductive age (15–44 years) and peri-menopausal age (45–54 years) groups, whereas that in the underweight group was the highest in the post-menopausal age (55–64 years) group. In all age groups of males, the age-related BMI gain value was the highest in the underweight group.

The age-related BMI gain values of all individuals in the post-pandemic period decreased to 0.02 kg/m^2^/year for females and − 0.02 kg/m^2^/year for males (*p* < 0.001, Table 1). In the reproductive age (15–44 years) group, the value in the normal weight group was significantly reduced in the post-pandemic period (*p* < 0.001). The values in the underweight group also showed a decreasing trend, although no significant difference was detected. A similar trend was also observed in the other age groups of females. For male participants, the BMI gain values in all age and BMI groups were significantly reduced in the post-pandemic period, except in the 45–54 years overweight or obesity and 55–64 years underweight groups, which also showed reduced trends. According to the linear regression analysis of the change in age-related BMI gain from pre-pandemic to post-pandemic periods (Table 2), the coefficients of age and sex (female) were both significantly positive (B = 0.002, *p* = 0.017; and B = 0.031, *p* = 0.038; respectively). But BMI in 2015 was not statistically significant (B = -0.001, *p* = 0.553).

**Table 2:** Change in the age-related BMI gain before and after the COVID-19 pandemic

Variables	Unstandardized Coefficients		Standardized Coefficients	*p*-value
	B	Standard error	Beta	
**Age**	**0.002**	**0.001**	**0.017**	0.017*
**Sex (Female)**	**0.031**	**0.015**	**0.015**	0.038*
BMI in 2015	− 0.001	0.002	− 0.004	0.553

*BMI* Body mass index

*Statistically significant

## Discussion

This study demonstrated age-related BMI gains in all age and BMI strata in the pre-pandemic period in the Japanese population, which is consistent with a previous finding [2. ]. However, in the post-pandemic period, the age-related BMI gains decreased in all strata, although no significant difference was detected in several strata, which was inconsistent with the previous findings from other countries [7. , 8. ]. Rather than BMI values in 2015, younger age and male were shown to be associated with a decrease in age-related BMI gain from pre-pandemic to post-pandemic. The frequency of those who consumed alcohol several times a week decreased, although the frequency of drinking out remained unclear.

Pre-pregnancy underweight is known to adversely affect perinatal outcomes, preterm birth, and small for gestational age events [6. , 7. ]. Therefore, currently, a high prevalence of underweight females of reproductive ages (20.7% of females aged 20–29 years) is a significant problem in Japan [14. ]. Previously annual weight loss was significantly associated with spontaneous preterm birth [6. ]. The present study suggests an increased prevalence of annual weight loss in females of reproductive age was highest in 2021, after the pandemic. Thus, the lifestyle change caused by the COVID-19 pandemic might negatively affect perinatal outcomes unless the females are advised on optimal BMI gain [6. ].

The present study has several strengths. First, the age-related BMI gain values in Japanese were determined during the pre-pandemic period. Second, actual measurements of body weight were used in this study. Most of the previous studies were based on the data from the questionnaire and reported the increasing body weight or BMI after the lockdown of the COVID-19 pandemic, not comparing the increment to the age-related BMI gain [7. ]. However, the present results were analyzed differently and showed the influence on BMI more strictly, considering age-related BMI gain in the pre-pandemic period. Third, we successfully assessed the pandemic’s influence by age- and BMI-specific groups. Our study also had a few limitations, which are as follows: including only workers with 7 years of complete data may have resulted in some bias. In other words, the distinctive characteristics of workers whose data were incomplete for some reasons may limit the generalizability of this study; those who died or were unable to receive annual health checkups for several reasons, including severe illness or unemployment during the period, were not included in the analysis. However, following individuals over time to assess their measured weight changes helped improve internal validity. In addition, we could not collect data on the factors related to BMI, including diet styles, and physical activities. The difference between present and previous studies might also be associated with different races and cultures. Further studies are needed to determine the factors related to reducing age-related BMI gain detected in the post-pandemic period. Japan’s epidemic control could be one of those factors.

## Conclusions

Age-related weight gain in Japan was reduced after the COVID-19 pandemic. These effects could lead to a higher incidence of being underweight in females of reproductive age, which is related to adverse perinatal outcomes. The results would be helpful to future studies for appropriate intervention.

## Supplementary Information

**Additional file 1: Table S1.** The frequencies of overweight/obesity, normal-weight, underweight, and annual weight loss for individuals aged 15-44 years old.

## Data Availability

The raw data supporting the conclusions of this article will be made available by the authors upon reasonable request and with permission from the Central Clinic group.

## Abbreviations

COVID-19: Coronavirus disease 2019
BMI: Body mass index
