# Supplementary material for: Trend changes in age-related body mass index gain after coronavirus disease 2019 pandemic in Japan: a multicenter retrospective cohort study
Source: Reprod Biol Endocrinol. 2023 Jan 19;21:7. doi: 10.1186/s12958-023-01061-1 (PMC9850706; doi:10.1186/s12958-023-01061-1)
Supplement: Supplementary file 1 — Additional file 1: Table S1. The frequencies of overweight/obesity, normal-weight, underweight, and annual weight loss for individuals aged 15-44 years old. [file 12958_2023_1061_MOESM1_ESM.docx]

Table S1. The frequencies of overweight/obesity, normal-weight, underweight, and annual weight loss for individuals aged 15-44 years old

1. Females

| Year | 2015 | 2016 | 2017 | 2018 | 2019 | 2020 | 2021 |
| --- | --- | --- | --- | --- | --- | --- | --- |
| Overweight/obesity | 12.2% | 12.8% | 13.9% | 14.6% | 15.5% | 16.7% | 16.8% |
| Normal-weight | 67.8% | 68.0% | 68.5% | 68.1% | 67.8% | 67.4% | 68.0% |
| Underweight | 20.0% | 19.2% | 17.6% | 17.3% | 16.8% | 15.9% | 15.2% |
| Weight loss | - | 38.7% | 39.0% | 42.0% | 39.9% | 42.1% | 43.6% |

1. Males

| Year | 2015 | 2016 | 2017 | 2018 | 2019 | 2020 | 2021 |
| --- | --- | --- | --- | --- | --- | --- | --- |
| Overweight/obesity | 26.6% | 27.1% | 29.5% | 30.7% | 32.2% | 33.7% | 33.9% |
| Normal-weight | 67.6% | 67.7% | 65.5% | 64.7% | 63.6% | 62.6% | 62.3% |
| Underweight | 5.9% | 5.2% | 5.0% | 4.6% | 4.2% | 3.7% | 3.8% |
| Weight loss | - | 41.1% | 38.2% | 39.8% | 42.1% | 40.4% | 47.4% |
